# Patterns, Cost, and Immunological Response of MDR vs. Non MDR-Bacteremia: A Prospective Cohort Study

**DOI:** 10.3390/pathogens12081044

**Published:** 2023-08-15

**Authors:** Georgios Schinas, Katerina Skintzi, Anne-Lise De Lastic, Maria Rodi, Charalambos Gogos, Athanasia Mouzaki, Karolina Akinosoglou

**Affiliations:** 1 School of Medicine, University of Patras, Rion, 26504 Patras, Greece; georg.schinas@gmail.com (G.S.); katrinski80@gmail.com (K.S.); cgogos@upatras.gr (C.G.); mouzaki@upatras.gr (A.M.); 2 Laboratory of Immunohematology, Division of Hematology, Department of Internal Medicine, Medical School, University of Patras, Rion, 26504 Patras, Greece; delastic@gmail.com (A.-L.D.L.); marodi_biol@yahoo.gr (M.R.); 3 Department of Internal Medicine and Division of Infectious Diseases, University General Hospital of Patras, Rion, 26504 Patras, Greece

**Keywords:** antimicrobial resistance, multidrug-resistant bacteremia, immunological response, clinical outcomes, healthcare costs

## Abstract

Background: Antimicrobial resistance (AMR) is a significant global health concern, posing a critical challenge for the effective management of infectious diseases. This study aimed to compare the immunological response, clinical outcomes, and associated costs in patients with bacteremia due to antibiotic-resistant vs. susceptible bacterial microorganisms. Methods: This study was a single-center, prospective cohort study conducted from May 2017 to November 2019. The study population consisted of patients admitted with a confirmed diagnosis of bacteremia. Results: A total of 116 patients were included, with 53 (45.7%) harboring non-multidrug-resistant (non-MDR) bacterial isolates and 63 (54.3%) harboring multidrug-resistant (MDR) bacterial isolates. Patients with MDR bacteremia had more severe clinical presentations, as indicated by higher SOFA and APACHE II scores. Results revealed higher all-cause mortality rates (39.7% vs. 17%) and median healthcare costs (€4791 vs. €2843.5) in the MDR bacteremia group. Moreover, MDR bacteremia was linked to higher levels of TNF-a, indicating a differential immune response. Furthermore, MDR bacteremia was found to be an independent predictor of mortality (OR = 3.216, 95% CI: 1.338–7.730, *p* = 0.009) and increased healthcare costs (effect size of approximately 27.4%). Conclusion: These findings underscore the significant impact of antimicrobial resistance in healthcare settings, highlighting the urgency of addressing the challenges posed by MDR microorganisms.

## 1. Introduction

The continuous emergence and rapid dissemination of antibiotic-resistant bacteria has become a significant public health concern worldwide [1], posing a critical challenge for the effective management of infectious diseases. According to the World Health Organization, antimicrobial resistance (AMR) is one of the biggest threats to global health, food security, and development today. Antibiotic-resistant infections kill at least 700,000 people worldwide each year, and this number is expected to rise to 10 million by 2050 [2].

In a clinical setting, infections caused by antibiotic-resistant pathogens are associated with increased morbidity, mortality, and healthcare costs compared to infections caused by antibiotic-susceptible microorganisms [3]. The immunological response of the host plays a vital role in the outcome of bacterial infections, yet the differences in host responses to antibiotic-resistant and susceptible bacteria remain poorly understood. Several factors may contribute to the differences in host responses to antibiotic-resistant and susceptible bacteria, including the virulence of the bacterial strain and host genetic factors [4,5]. Notably, emerging evidence suggests that a compromised immune status may not pose a risk factor for multi-drug resistant (MDR) bacterial infection as previously thought [6]. In fact, the immunosuppressed status of any kind may offer protection against MDR colonization in an intensive care unit (ICU) environment [7]. What is more, a robust and sustained immune response may also play a crucial role in preventing the emergence of new antibiotic-resistant strains [8]. Therefore, understanding the host response to antibiotic-resistant bacterial infections is important both for developing novel therapeutic strategies and optimizing the clinical management of these infections.

The escalating crisis of AMR in healthcare systems has also led to increased hospitalization durations, healthcare-associated costs, and the need for more aggressive and expensive treatment options [9]. This heightened urgency for innovative therapies and preventive measures has fueled research into new antimicrobial agents, alternative treatment strategies, rapid diagnostic tests, and targeted immunotherapies [10]. Furthermore, the financial pressure imposed on institutional and national budgets has encouraged multidisciplinary collaboration among researchers, clinicians, and policymakers to optimize antimicrobial stewardship programs, enhance surveillance networks, and promote public awareness campaigns [11]. Ultimately, the comprehensive approach driven by the economic implications of AMR aims to mitigate the spread of resistant infections, reduce healthcare costs, and improve patient outcomes [12].

The present study aims to compare the immunological response, as reflected in the levels of significant pro- and anti-inflammatory cytokines, investigate possible host or infection factors that determine it, study the corresponding clinical outcomes, and evaluate the cost associated with antibiotic treatment and hospitalization in patients with bacteremia due to antibiotic-resistant vs. susceptible bacterial microorganisms.

## 2. Materials and Methods

### 2.1. Study Design and Setting

This was a single-center, prospective cohort study conducted at the University General Hospital of Patras, a tertiary care, academic hospital in the region of Western Greece. Patients admitted to the hospital with a diagnosis of bacteremia between May 2017 and November 2019 were consecutively recruited. Subsequently, patients were divided into two groups based on the isolated microorganism’s resistance pattern. The study was approved by the hospital’s ethics committee (No 67/15.04.2016), and informed consent was obtained from all participating patients.

### 2.2. Study Population and Inclusion/Exclusion Criteria

Patients admitted to the University General Hospital of Patras with a confirmed diagnosis of bacteremia (primary or secondary) were eligible for inclusion in the study. Inclusion criteria were (1) admission to the hospital with a diagnosis of infection of any focus, (2) microbiologically confirmed bacteremia, and (3) written informed consent. Exclusion criteria included evidence of blood culture contamination, the presence of more than one microorganism, relapsing bacteremia, and the inability to provide written informed consent.

### 2.3. Data Collection and Clinical Outcomes

Upon obtaining consent, anonymized and coded data on epidemiological (including gender, age, and comorbidities) and clinical characteristics, laboratory values (on admission day), and administered antibiotic treatment (throughout hospitalization) were recorded. The severity scoring systems (APACHE II, SAPS II, SOFA score) were evaluated at the time of positive blood culture. Information related to outcomes, including all-cause mortality, treatment success rate (defined as having at least one confirmed follow-up blood culture without the presence of the infecting bacteria), length of hospital stay (LOS), need for surgery, and changes in antibiotic regimens, was also documented. In our analysis of the LOS, we included all patients, regardless of their survival status. The cost of hospitalization was calculated based on the list of “Closed Consolidated Hospitalizations” as defined for Greek hospitals—ΚΕΝ (Kλειστά Ενοποιημένα Nοσήλια) in Greek—and for each infection based on the ICD10 coding for infection-related entities. The cost of antimicrobial treatment was calculated separately for each patient based on the hospital price of the antimicrobial unit, the number of days of use, and the total units received by the hospitalized patient.

### 2.4. Antimicrobial Resistance Pattern Definitions

When a patient’s bacteremia was attributed to recent hospitalization (up to 90 days prior to the current one), we classified this case as a healthcare-associated case. We extended this classification to include patients who reside in healthcare or eldercare facilities, senior living communities, or retirement homes. All other instances were classified as community-onset cases. We grouped the isolates that displayed either intermediate antibiotic susceptibility or resistance into a collective category we referred to as the non-susceptible group and we classified an isolate as non-susceptible to an antimicrobial group if it displayed non-susceptibility to even one agent within the group. As such, each isolate was distributed in one of the two following categories: (a) Non-MDR (nMDR): These are isolates that were non-susceptible to no more than two antimicrobial groups. (b) MDR: These are isolates that were non-susceptible to three or more antimicrobial groups but still susceptible to at least two groups.

### 2.5. Blood Sampling and Immunological Analysis

Upon the detection of bacteremia, blood samples (5 mL) were collected from a peripheral vein into BD Vacutainer SSTII Serum Separator Tubes (cat#367955). The tubes were gently inverted 4–5 times, subsequently left to clot for 30 min, and centrifuged for 10 min at 1600× *g* at room temperature. The serum was aliquoted and stored at −80 °C until needed. Measurement of the concentration of the cytokines IL-1b, IL-6, IL-8, IL-10, IL-12p80, and TNF in patient serum samples was performed with a BD FACS Array Bioanalyzer, using a cytometric bead array (CBA).

### 2.6. Statistical Methodology

The primary objectives of this study were to evaluate the impact of MDR bacteremia on patient outcomes, quantify the economic burden of disease management in the hospital setting, and assess the accompanying immunological response of the host. To achieve these, we first conducted a comparative descriptive analysis of the study cohort and subsequently performed a comprehensive statistical analysis based on our observations utilizing regression analysis.

Descriptive statistics were employed to summarize and present the demographic and clinical characteristics of the study population. Continuous variables were reported as medians with interquartile ranges (IQR), while categorical variables were expressed as counts and percentages. The Shapiro–Wilk test was used to assess the normality of the data distribution. The comparative analysis between non-MDR and MDR patients was performed using either Student’s *t*-test or the Mann–Whitney U test for continuous variables and the Chi-square or Fisher’s exact test, as appropriate, for categorical variables. A *p*-value of <0.05 was considered statistically significant. As most variables were not normally distributed, non-parametric statistical methods were employed for subsequent analyses. Spearman’s rank correlation was performed to determine the correlations between variables. Correlation coefficients were interpreted using standard guidelines, where the strength of correlation was defined as weak (0.1–0.3), moderate (0.3–0.5), and strong (>0.5).

To further explore the relationship between MDR status and the total cost of hospitalization, we utilized a Generalized Linear Model (GLM) with a gamma distribution and a log link function. The decision for this model was made based on the positive skewness and non-negative nature of our dependent variable, the total cost of hospitalization. The model was adjusted for potential confounding variables including age, sex, length of hospital stay (LOS), and the Charlson Comorbidity Index (CCI). The statistical significance of each predictor was assessed using the Wald Chi-Square test. The Akaike’s Information Criterion (AIC) helped us ensure that our model was well-suited to the data.

To identify independent predictors of mortality among patients with bacteremia, regression analysis was employed. First, univariate logistic regression analyses were conducted to evaluate the influence of each independent variable (age, gender, BMI, MDR status, and CCI score) on mortality. The results were presented as odds ratios (ORs) along with their 95% confidence intervals (CIs) and corresponding *p*-values. Subsequently, we built a multivariate model using only significant predictors. The results were presented as adjusted ORs along with their 95% CIs and corresponding *p*-values.

All statistical tests were two-sided, and a *p*-value of less than 0.05 was considered statistically significant. All statistical analyses were performed using the Statistical Package for the Social Sciences (SPSS) version 28.0 (IBM Corp., Armonk, NY, USA).

## 3. Results

### 3.1. Cohort Characteristics

The comparison between patients with non-multidrug-resistant (non-MDR) and multidrug-resistant (MDR) bacteremia is coherently presented in Table 1.

A total of 116 patients were included, with 53 (45.7%) harboring non-MDR bacterial isolates and 63 (54.3%) harboring MDR bacterial isolates. The median age of the patients was 72 years (IQR 60–83) in both the non-MDR and MDR groups, with no significant differences in sex, age, or body mass index (BMI) between the groups. Patients with MDR bacteremia had a significantly higher Charlson’s Comorbidity Index (CCI) score compared with those with non-MDR bacteremia, although median values were the same for both subgroups (median 5 [IQR 4–7] vs. 5 [IQR 3–6], *p* = 0.016). The proportion of hospital-acquired bacteremia was significantly higher among MDR patients (57.1% vs. 22.6% for non-MDR, *p* < 0.001). The presence of ESKAPE (*Enterococcus faecium*, *Staphylococcus aureus*, *Klebsiella pneumoniae*, *Acinetobacter baumannii*, *Pseudomonas aeruginosa*, and *Enterobacter* species) isolates was higher in the MDR group, although not statistically significant (82.5% vs. 67.9% for non-MDR, *p* = 0.067). In contrast, coagulase-negative staphylococci (CNS) isolates were significantly more prevalent among MDR-bacteremia patients (13.2% vs. 0% for non-MDR, *p* = 0.004).

### 3.2. Disease Severity Is Associated with Presence of MDR Pathogens

Patients with MDR bacteremia had a marginally significantly higher Sequential Organ Failure Assessment (SOFA) score (*p* = 0.049) and Acute Physiology and Chronic Health Evaluation II (APACHE II) score (*p* = 0.005) than those with non-MDR bacteremia. The incidence of septic shock was also significantly higher in MDR patients (17.5% vs. 3.8% for non-MDR, *p* = 0.02). Laboratory values revealed significantly lower white blood cell (WBC) counts among MDR-bacteremia patients (*p* = 0.036) (Table 1)

### 3.3. Disease Severity Is Associated with Underlying Inflammatory Response

APACHE II showed a statistically significant positive correlation with the concentrations of IL-6, IL-8, and IL-10 (rho = 0.295, *p* < 0.01; rho = 0.375, *p* < 0.01; rho = 0.274, *p* < 0.01, respectively). SOFA also demonstrated a significant positive correlation with IL-6, IL-8, and IL-10 concentrations (rho = 0.261, *p* < 0.01; rho = 0.214, *p* < 0.05; rho = 0.234, *p* < 0.05, respectively). SAPS II displayed significant positive correlation with IL-6, IL-8, and IL-10 (rho = 0.379, *p* < 0.01; rho = 0.372, *p* < 0.01; rho = 0.390, *p* < 0.01, respectively). These findings suggest that as the severity of the disease increases, as indicated by higher APACHE II, SOFA, and SAPS II scores, there is a corresponding increase in these three interleukin concentrations, although the strength of associations based on their coefficients may be considered weak to moderate.

### 3.4. Inflammatory Response Varies between MDR and Non-MDR Bacteremias

In terms of the inflammatory response, the MDR subgroup had significantly higher levels of TNF-a (*p* = 0.03), whereas no significant differences were observed for the other investigated interleukins (IL-1β, IL-6, IL-8, IL-10, and IL-12p70) between the subgroups.

### 3.5. Clinical Outcomes Are Worse in MDR Bacteremia

Regarding the recorded clinical outcomes, the success of treatment in patients with non-MDR bacteremia was calculated at 37.7% (20 out of 53 patients), compared to 22.2% (14 out of 63 patients) in MDR bacteremia patients. However, this difference did not reach statistical significance (*p* = 0.06). The requirement for a change in antibiotic therapy was comparable between the two subgroups, with 34% (18 out of 53) of non-MDR bacteremia patients and 37% (23 out of 63) of MDR bacteremia patients necessitating a change, a difference that was not statistically significant (*p* = 0.77). Surgery was needed significantly less in the MDR bacteremia group, where only 1.6% (1 out of 62) underwent surgery compared to 11.3% (6 out of 53) in the non-MDR bacteremia group (*p* = 0.028). In terms of the length of stay (LOS), there was a noticeable difference between patients with non-MDR and MDR bacteremia, although not statistically significant (13 days [IQR 8–24] vs. 19 days [IQR 9–32], *p* = 0.06). Finally, the all-cause mortality rate was significantly higher in the MDR bacteremia group at 39.7% (25 out of 62) compared to 17% (9 out of 53) in the non-MDR bacteremia group (*p* = 0.007).

### 3.6. MDR Bacteremia Is an Independent Predictor of Mortality

Age, gender (female), MDR status, CCI score, and various severity indices (SAPS II, APACHE II, and SOFA scores) were analyzed for their correlation with the outcome of death. The results are presented in Table 2 for both univariate analysis and the constructed multivariate models. The univariate analysis revealed that an individual’s MDR status (OR = 3.216, 95% CI: 1.338–7.730, *p* = 0.009) and CCI score (OR = 1.328, 95% CI: 1.095–1.611, *p* = 0.004) significantly affected the likelihood of death. This was not the case for age (OR = 1.019, 95% CI: 0.991–1.047, *p* = 0.182) and female sex (OR = 1.368, 95% CI: 0.607–3.084, *p* = 0.449), which were not significantly associated with death.

### 3.7. Healthcare-Related Costs Are Higher in MDR Bacteremia

From an economic perspective, the costs associated with hospitalization showed a significant disparity between the two groups. Specifically, the antibiotic costs for patients with MDR bacteremia were significantly higher than those with non-MDR bacteremia (median €1571 [IQR 592–4599] vs. €498.4 [IQR 201–1457], *p* < 0.001). Although the hospitalization costs showed an increase in the MDR group, the difference was not statistically significant (median €3840 [IQR 1574–6698] vs. €2150 [IQR 1421–3710], *p* = 0.078). However, when considering the total cost of care, incorporating both hospitalization and antibiotic costs, the difference was statistically significant (median €4791 [IQR 2194–12,468] vs. €2843.5 [IQR 1721–5165], *p* = 0.005), while antibiotic costs in the case of non-MDR pathogens represent 17.5% of the total cost of care vs. 32.7% in the case of MDR, suggesting a substantial economic burden associated with MDR bacteremia.

### 3.8. MDR Bacteremia Presents an Independent Predictor of Increased Healthcare-Related Cost

Moreover, we conducted a comprehensive analysis of hospitalization costs related to MDR bacteremia using a Generalized Linear Model (GLM). This model was equipped with a gamma distribution and log link, taking into account patients’ age, sex, length of stay (LOS), Charlson Comorbidity Index (CCI), and MDR status as contributing factors. MDR status and LOS were identified as statistically significant determinants of the total cost of hospitalization within the context of bacteremia. (*p* = 0.003 and *p* < 0.001, respectively). Patients with MDR bacteremia were associated with a 0.327 unit increase in the log-transformed total cost, compared to those with non-MDR bacteremia (*p* = 0.003). This translates to an effect size of approximately 27.4%, indicating that when compared to non-MDR bacteremia patients, MDR bacteremia patients tend to have higher overall hospitalization costs. With regards to LOS, each additional day in the hospital led to a 0.040 unit increase in the log-transformed total cost, or an effect size of approximately 4.08%. On the other hand, age, sex, and the Charlson Comorbidity Index (CCI) were not significant predictors of the total hospitalization cost (*p* = 0.495, *p* = 0.817, and *p* = 0.467, respectively).

**Figure 1:**
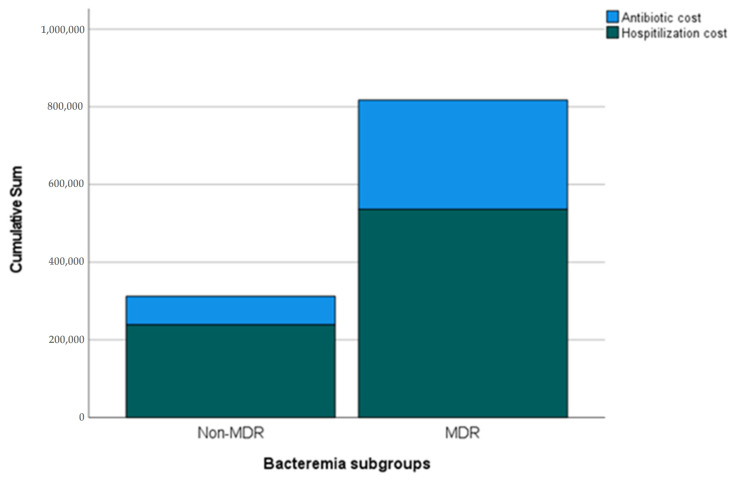
Distribution of Total and Antibiotic Costs between Non-MDR and MDR Bacteremia. Each bar represents a category of bacteremia (Non-MDR and MDR) and is divided into two segments: The lower segment indicates the total hospitalization costs excluding antibiotic expenses, while the upper segment illustrates the antibiotic-associated costs specifically. Sum indicates cost in Euros (€).

Figure 1 illustrates the distribution of costs between non-MDR and MDR bacteremia cases, highlighting the comparative economic burden of non-MDR vs. MDR bacteremia, emphasizing the increase in both hospitalization costs and the proportion of costs specifically tied to antibiotic treatment.

## 4. Discussion

In summary, our results indicate that patients with MDR bacteremia exhibit more severe clinical presentation, worse outcomes, and higher healthcare costs than those with non-MDR bacteremia. Furthermore, disease severity seems to be interlinked with both the presence of MDR pathogens and the differential expression of the underlying inflammatory response. Although not significantly different, the levels of pro- and anti-inflammatory mediators underlying the inflammatory response appear to vary between MDR and non-MDR bacteremia.

In our study, disease severity and worse clinical outcomes were associated with the presence of MDR bacteremia. Moreover, MDR bacteremia was found to be an independent predictor of mortality. In contrast to widely accepted beliefs, the clinical implications of infections caused by MDR pathogens have been the subject of ongoing debate in the medical community. Although it is generally acknowledged that MDR infections are linked to longer hospital stays, there is still controversy surrounding the potential association between antimicrobial resistance and mortality rates. Indeed, research on antimicrobial resistance in the ICU setting has produced varied and sometimes conflicting results regarding its association with patient outcomes. The Extended Prevalence of Infection in Intensive Care (EPIC II) study, involving 1265 ICUs from 76 countries, found that antimicrobial resistance in the ICU does not consistently lead to the worst outcomes [13]. Additionally, a literature review of 24 studies conducted on ICU populations worldwide yielded inconsistent results regarding the impact of MDR infections on mortality [14]. Further evidence from studies such as that conducted by Blot et al. suggests that antibiotic resistance in nosocomial bacteremia caused by Gram-negative bacteria may not adversely affect the outcomes of critically ill patients [15]. Similarly, a Finnish cohort study by Kontula et al. indicated that the causative pathogens of nosocomial bloodstream infections, including MDR bacteria, may not necessarily be interpreted as a risk factor for severe outcomes in patients [16]. However, a recent meta-analysis consistently demonstrated notable patterns indicating higher mortality rates and increased resource utilization, encompassing extended hospital stays and higher direct costs associated with healthcare-associated infections of MDR microorganisms compared with infections linked to susceptible microorganisms [17]. Specifically, the authors observed a 1.27-fold increase in excess length of stay, a 1.33-fold increase in costs, and a 1.62-fold increase in the risk of mortality at discharge associated with MDR infections [17]. The reasons for these discrepancies include diverse study designs, MDR and outcome definitions, variable sites of infection, the population and pathogen studied, as well as the presence of an adequate control group, while in most cases, studies had small sample sizes. Interestingly, studies examining the microbial genome have indicated that bacterial genotyping may be more predictive of adverse infection outcomes and mortality than other factors such as patient age, sex, and comorbidities [18,19]. This suggests that virulence, rather than resistance alone, may play a crucial role in determining the lethal effects of infections.

The severity and outcome of the disease were also linked to the inflammatory response and cytokine levels. Numerous studies have indicated elevated cytokine levels in patients with septic shock and a correlation between cytokines and non-survivors, as well as severity scores [20,21,22,23]. Consistent with these findings, the patients with more severe disease in our study cohort exhibited higher levels of IL-6, IL-8, and IL-10.

The inflammatory response seemed to vary between MDR and non-MDR bacteremia, regarding TNF-a, while no other significant differences in other cytokine levels were detected. Prior investigations conducted in animal models have revealed variations in the immune response and virulence among isolates with distinct susceptibility patterns. Giamarellos-Bourboulis et al. conducted a study that demonstrated that rabbits infected with susceptible *P. aeruginosa* exhibited higher TNF-α levels and reduced survival than those inoculated with MDR strains [24]. Similarly, Karamouzos et al. observed significantly lower levels of the potent pro-inflammatory cytokine TNF-α in patients with sepsis caused by resistant pathogens [25]. In the vitreous of patients with distinct clinical characteristics, an intensified inflammatory response involving IL-10, IL-6, IL-8, IL-1β, and TNF-α was observed in cases of MDR *P. aeruginosa* endophthalmitis compared to infections caused by sensitive *P. aeruginosa* [26]. Research findings have consistently demonstrated increased levels of IL-10 in cases of MDR *P. aeruginosa*-induced sepsis compared to non-MDR *P. aeruginosa* infection in sepsis patients and in a mouse model of *P. aeruginosa* pneumonia [27,28]. These findings align with previous observations in conditions such as MDR tuberculosis (MDR-TB), acute organ dysfunction, and bacteremia, where elevated IL-1β concentrations were observed in MDR-TB [29], as well as in MDR Escherichia coli and Klebsiella infections [30]. Similarly, Zhou et al., using three different isolates of K. pneumoniae, found that pan-drug-resistant isolates elicited a lower inflammatory response compared to MDR and susceptible isolates [31]. Furthermore, Basingnaa et al. reported that the mean levels of IL-10 and TNF-α in MDR-TB cases were relatively higher than those in drug-susceptible tuberculosis cases [32]. Several studies have also reported a correlation between elevated IL-6 levels and susceptibility to pathogens and disease severity in patients with human immunodeficiency virus and other experimental infections [27,33,34,35]. Recent studies on inflammatory responses have shown discrepancies. The sample sizes remain small and are heterogeneous in terms of the pathogens involved. Additionally, the comorbidities of the populations studied and variability in clinical settings hinder the drawing of solid conclusions. Nonetheless, it has now become evident that the induced immune response may differ based on the susceptibility patterns of pathogens. The well-characterized mutations that lead to antibiotic resistance are now revealing a new aspect: A dual function, in which these mutations also have an impact on immune effector mechanisms. This emerging evidence suggests that antibiotic resistance mutations not only protect bacteria from the effects of antibiotics but also have additional effects on how the immune system responds to infection and accomplishes immune evasion [4]. Studies have shown that the acquisition of certain point mutations in specific genes of daptomycin-resistant methicillin-resistant Staphylococcus aureus leads to a notable decrease in the secretion of proinflammatory cytokines, such as TNF-α, interleukin-6 (IL-6), and macrophage inflammatory protein-1β (MIP-1β), along with the attenuated expression of IL-1β, TNF-α, CXCL2, and CCL2 in MRSA biofilms [36,37]. This dual effect of antibiotic resistance mutations, which influences both the bacterial response to antibiotics and the modulation of immune effector mechanisms, highlights the intricate interplay between microbial resistance and the host immune response.

Healthcare-related costs were higher in bacteremia-caused MDR pathogens, whereas MDR bacteremia was an independent predictor of increased healthcare-related costs in our study. The cost in a real-world setting was previously explored by our group [38,39]. It appears that successful empirical antibiotic treatment lowers healthcare-related costs [39], even though the cost varies with the site of infection [38]. However, in a setting of high AMR, antibiotic therapy commonly fails [40], leading to significantly extended hospital stays, more frequent visits to doctors, and longer recovery periods, resulting in a higher prevalence of long-term disability [41]. Research has shown that patients with antibiotic-resistant infections experience hospital stays that are prolonged by 6.4–12.7 days, collectively contributing an additional eight million hospital days. The medical cost per patient afflicted with an antibiotic-resistant infection varies from $18,588 to $29,069 according to estimates [40,42]. Moreover, the overall economic impact on the U.S. economy due to antibiotic-resistant infections is substantial, with estimated healthcare costs reaching as high as $20 billion, and an additional $35 billion per year attributed to lost productivity. This burden also extends to families and communities that face financial challenges due to lost wages and increased healthcare expenses [40,41]. In a more recent CDC retrospective cost analysis of patients with six common resistant infections in the Veterans Health Administration medical centers, the direct estimated cost was $4.6 billion per year [43]. It is important to highlight that the scope of the report was limited to presenting only the direct medical costs related to a positive culture for the specific pathogens under investigation. Consequently, downstream costs associated with potential long-term disabilities resulting from antibiotic-resistant infections were not included in the analysis. Additionally, the report did not account for the financial impact on patients due to missed work and other related expenses nor did it consider the post-discharge costs borne by the health system. It is crucial to recognize that the full economic burden of antibiotic-resistant infections extends beyond the direct medical costs discussed in this report. The long-term consequences and potential disabilities that patients may experience after hospitalization can lead to additional financial strain on individuals, families, and society as a whole. Moreover, the cost to the health system may increase significantly if patients require ongoing care and treatment following discharge. The impact of resistance on the quality of life was even more pronounced. According to the OECD models, which use disability-adjusted life years (DALYs) as an indicator, taking into account premature deaths and time spent in a state of compromised health, approximately 1.75 million years of healthy life are lost annually across 33 countries [44].

Our study had several limitations. First, it was a single-center study with a limited number of patients. The small size did not allow for further inflammatory response profile differentiation between various pathogens, which could also control for variable pathogen virulence. Despite our efforts to include all consecutive bacteremia cases, potential selection bias inherent in prospective cohort studies cannot be completely ruled out. Moreover, our study used a single time-point sampling method. However, the inflammatory response is a dynamic and constantly evolving process. Serial measurements and studies of temporal trends may be more accurate in examining associations with MDR pathogens and their respective outcomes. In addition, the inflammatory response may vary depending on the site of infection. It is reasonable to hypothesize that deep-seated infections increase a different inflammatory response compared to that of hollow viscous fluid. Furthermore, it should be noted that the source of bacteremia may significantly influence LOS. Additionally, factors such as the appropriateness of the initial antimicrobial therapy, potential administration of immunomodulatory regimens including antibiotics with respective properties (e.g., macrolides and quinolones), chronic underlying diseases, the proportion of secondary bacteremia, and variations in source control measures, including surgical interventions, could have introduced bias in terms of the assessment of the underlying inflammatory response and outcomes studied. In our study, we used the APACHE II, SAPS II, and SOFA scores to assess severity, which is widely accepted and commonly used in a clinical setting, but we acknowledge that we did not use a bacteremia-specific scoring system. Last, as previously well stated, the quantification of costs related to MDR is far from an easy task since indirect costs extending far beyond hospitalization and including further complications of colonization or rehabilitation are to be considered.

To conclude, our study provides evidence that patients with MDR bacteremia exhibit a more severe clinical presentation, worse outcomes, and higher healthcare costs than those with non-MDR bacteremia. Furthermore, disease severity seems to be interlinked with both the presence of MDR pathogens and differential expression of the underlying inflammatory response. Our findings underscore the significant impact of antimicrobial resistance in healthcare settings and highlight the urgent need to address the challenges posed by MDR microorganisms. The increased burden on patients, healthcare facilities, and the overall healthcare system necessitates a comprehensive approach to tackle the problem of AMR effectively. By recognizing and understanding these patterns, healthcare providers and policymakers can develop targeted strategies to mitigate the adverse effects of MDR infections and improve patient outcomes.

## Figures and Tables

**Table 1:** Comparative Assessment of Epidemiological, Clinical, Economic, and Immunological Characteristics in Patients Harboring non-MDR vs. MDR Bacterial Blood Isolates.

Parameters	Non-MDR (n = 53)	MDR (n = 63)	*p*-Value
Age (years)	72 (60–83)	72 (59–83)	0.78
Male Sex (%)	32 (60.4)	39 (61.9)	0.86
BMI (kg/m^2^)	24.9 (23.4–26)	24.8 (23–25.7)	0.81
CCI Score	5 (3–6)	5 (4–7)	0.016
Hospital Acquired (%)	12 (22.6)	36 (57.1)	<0.001
	**Isolated pathogen**		
Gram (+) (%)	18 (34)	21 (33.3)	0.94
Gram (−) (%)	35 (66)	42 (66.7)	0.94
*Staphylococci* (%)	10 (18.9)	15 (23.8)	0.51
*Enterobacterales* (%)	24 (45.3)	19 (30.2)	0.09
*Pseudomonas aeruginosa* (%)	9 (17)	8 (12.7)	0.51
** Clinical Severity **			
SOFA Score	4 (2–5)	5 (3–7)	0.049
APACHE II Score	14 (10–17)	17 (13–21)	0.005
SAPS II Score	33 (27–40)	37 (29–46)	0.14
Septic Shock (%)	2 (3.8)	11 (17.5)	0.02
AKI (%)	9 (17)	4 (6.3)	0.07
** Laboratory Values **			
WBC (×10^3^/µL)	13,620 (8805–15,960)	9940 (4740–15,090)	0.036
PMN (%)	81.9 (78.1–90.2)	82.2 (69–89.9)	0.12
Lymph (%)	7.5 (5.05–13.35)	10.6 (4.6–15.75)	0.22
Mono (%)	5.7 (2.95–8.35)	6.7 (3–9.2)	0.44
ESR (mm/h)	70 (49.25–102.25)	80 (46–104.5)	0.6
CRP (mg/L)	19.05 (9.78–29.52)	10.7 (7.44–23.3)	0.12
Immunoglobulins (g/L)	2.94 (2.5–3.4)	2.7 (2.42–3.1)	0.24
** Inflammatory response **			
IL-1β (pg/mL)	1.45 (0.58–2.21)	1.42 (0.8–2.25)	0.86
IL-6 (pg/mL)	56.1 (31.48–106.55)	73.79 (27.55–223.9)	0.19
IL-8 (pg/mL)	123.5 (52.34–227.45)	131.7 (71.43–210.35)	0.59
IL-10 (pg/mL)	3.19 (1.6–5.8)	3.89 (1.58–7.58)	0.44
IL-12p70 (pg/mL)	2.61 (2.11–4.35)	3.68 (2.38–4.68)	0.052
TNF-a (pg/mL)	1.52 (1.08–4.42)	3.91 (1.41–4.96)	0.03
** Clinical Outcomes **			
Treatment success (%)	20 (37.7)	14 (22.2)	0.06
Change of Antibiotic Therapy (%)	18 (34)	23 (36.5)	0.77
Surgery (%)	6 (11.3)	1 (1.6)	0.028
Death (%)	9 (17)	25 (39.7)	0.007
Length of Stay (Days)	13 (8–24)	19 (9–32)	0.06
	** Hospitalization-associated Costs **		
Antibiotic Cost (Euro)	498.4 (201–1457)	1571 (592–4599)	<0.001
Hospitalization Cost (Euro)	2150 (1421–3710)	3840 (1574–6698)	0.078
Total Cost (Euro)	2843.5 (1721–5165)	4791 (2194–12,468)	0.005
❖ Continuous variables are expressed as median (interquartile range) and categorical variables are expressed as count (percentage). ❖ The *p*-values in this table were calculated using the Mann–Whitney U test for continuous variables and Chi-square or Fisher’s exact test for categorical variables as appropriate. ❖ *p*-values less than 0.05 were considered statistically significant. ❖ Non-MDR refers to non-multidrug-resistant, while MDR refers to multidrug-resistant. ❖ Abbreviations: MDR—Multi-Drug Resistant; BMI—Body Mass Index; CCI—Charlson Comorbidity Index; SOFA—Sequential Organ Failure Assessment; APACHE II—Acute Physiology and Chronic Health Evaluation II; SAPS II—Simplified Acute Physiology Score II; AKI—Acute Kidney Injury; WBC—White Blood Cells; PMN—Polymorphonuclear Neutrophils; ESR—Erythrocyte Sedimentation Rate; CRP—C-reactive protein; IL—Interleukin; TNF-a—Tumor Necrosis Factor-alpha.			

**Table 2:** Univariate and Multivariate Analysis of Factors Correlated with Mortality.

Variable	Odds Ratio	95% Confidence Interval	*p*-Value	Adjusted Odds Ratio	95% Confidence Interval	*p*-Value
Age	1.019	0.991–1.047	0.182			
Sex	1.368	0.607–3.084	0.449			
Hospital-acquired	1.950	0.868–4.382	0.106			
BMI	1.035	0.896–1.194	0.642			
MDR	3.216	1.338–7.730	0.009	2.74	1.09–6.85	0.031
CCI	1.328	1.095–1.611	0.004	1.26	1.02–1.55	0.032

## Data Availability

Data can be made available following a reasonable request to the corresponding author.
